# Investigation of Photon Radiation Attenuation Capability of Different Clay Materials

**DOI:** 10.3390/ma14216702

**Published:** 2021-11-07

**Authors:** Mohamed Elsafi, Yousry Koraim, Mansour Almurayshid, Fahad I Almasoud, M. I. Sayyed, I. H. Saleh

**Affiliations:** 1 Physics Department, Faculty of Science, Alexandria University, Alexandria 21511, Egypt; 2 Department of Environmental Studies, Institute of Graduate Studies and Research, Alexandria University, Alexandria 21511, Egypt; yousrymasry92@gmail.com (Y.K.); igsr.ihindawy@alexu.edu.eg (I.H.S.); 3 Nuclear Science Research Institute (NSRI), King Abdulaziz City for Science and Technology (KACST), Riyadh 11442, Saudi Arabia; malmurayshid@kacst.edu.sa (M.A.); or fmasaud@gmail.com (F.I.A.); 4 Department of Soil Sciences, College of Food and Agricultural Sciences, King Saud University, Riyadh 12372, Saudi Arabia; 5 Department of physics, Faculty of Science, Isra University, Amman 11622, Jordan; mabualssayed@ut.edu.sa; 6 Department of Nuclear Medicine Research, Institute for Research and Medical Consultations (IRMC), Imam Abdulrahman Bin Faisal University (IAU), P.O. Box 1982, Dammam 31441, Saudi Arabia

**Keywords:** clay, radiation attenuation, WinXCom program, mass attenuation coefficient

## Abstract

This work aims to experimentally report the radiation attenuation factors for four different clays (red, ball, kaolin and bentonite clays) at four selected energies (emitted from Am-241, Cs-137, and Co-60). The highest relative difference in the mass attenuation coefficient (MAC) is equal to −3.02%, but most of the other results are much smaller than this value, proving that the experimental and theoretical data greatly agree with each other. From the MAC results, the shielding abilities of the clay samples at 0.060 MeV follow the order of: bentonite > red > ball > kaolin. Thus, at low energies, the bentonite clay sample provides the most effective attenuation capability out of the tested clays. The half value layer (HVL) increases as energy increases, which suggests that, only a thin clay sample is needed to sufficiently absorb the radiation at low energies, while at higher energies a thicker sample is needed to shield the same amount of high energy radiated. Furthermore, bentonite clay has the lowest HVL, while the kaolin clay has the greatest HVL at all energies. The radiation protection efficiency (RPE) values at 0.060 MeV are equal to 97.982%, 97.137%, 94.242%, and 93.583% for bentonite clay, red clay, ball clay, and kaolin clay, respectively. This reveals that at this energy, the four clay samples can absorb almost all of the incoming photons, but the bentonite clay has the greatest attenuation capability at this energy, while kaolin clay has the lowest.

## 1. Introduction

Radiation and nuclear technologies are widely applied in fields such as optimizing industrial processes, continuous form goods and products, nuclear power plants, radiology and nuclear medicine departments, and nuclear research and accelerator centers [1,2,3,4,5]. The harm caused by this technology is exposure to a high level of radiation to individuals, the public and the environment. Radiation protection aims to protect people and the environment from the harmful effects of ionizing radiation exposure, and shielding is the most effective factor of the radiation protection process, as it can decrease the intensity of the incident radiation. Hence, it is very important to determine radiation shielding parameters of different kinds of building material to assess their capability of attenuating ionizing radiation when they are used as building materials in places that contain radiation sources [6,7,8,9,10,11,12].

The MAC is a fundamental radiation shielding parameter defining the interaction of gamma rays within the material. This parameter is used to assess the capability of the material to attenuate gamma rays. Due to its high density, lead is commonly utilized as shielding material against gamma radiation; however, it has environmental toxicity and is expensive when used in large dimensions application. Concrete and other building materials are also used as radiation shielding materials [13,14,15]. Many studies have aimed at calculating the mass attenuation coefficients of different construction materials in order to evaluate their gamma ray attenuation abilities [16,17,18].

In the present study, some types of Egyptian clays which are considered as natural building material were investigated as radiation shielding material. The interest in studying those types of building material arises from their high chemical durability, weathering resistance, low hydraulic conductivity and relatively high hardness beside their natural availability. Clays have less resistance to chemical and environmental factors compared to other shielding materials, and are more available in Egypt [19,20].

The present clays are clean and environmentally friendly construction materials, can be used in radiation protection application as a radiation shield, and can be added to concrete mixes as an alternative to sand in certain proportions which lead to increase their density enhancing gamma ray attenuation. The high melting point of these clays indicates their thermal stability in case of long exposure to high energy radiation and their compressive strength is suitable for producing high-quality shielding materials. Thus, the determination of radiation shielding parameters of different types of clays is very useful.

The present work discusses the attenuation parameters for different clays, which is different from the previous work [21], which talks about improving bentonite clay with cement and not bentonite raw materials, and the results show a clear difference between the two studies. Additionally, in the research that studies the attenuation coefficient of both ball and kaolin clays [22], these clays were extracted from Nigeria, while our clays were extracted from Egypt, in addition to studying two other types of clay (bentonite and red clays), which gives an important overview to the reader as it is a combined materials study.

## 2. Materials and Methods

**Figure 1:**
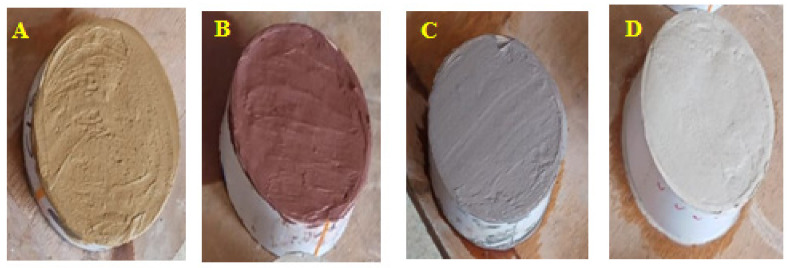
The different prepared clay samples.

Red, ball, kaolin, and bentonite are four types of clay that were obtained from quarries in Egypt’s Aswan, Abuznima, and Fayoum governments. They were stoned, ground to powder size, and sun-dried. The samples were sieved to 100 μm size, then they were thoroughly mixed with water, sectioned into sectors, and dried in the sun rays. Afterwards temperatures of 500 °C were used to bake the samples. Figure 1 illustrates bentonite, red, ball and kaolin clay samples labeled as A,B,C and D respectively. The porosity, P of the sample known as the ratio between the void volume and the total volume and can be calculated using the following equation [23,24].

(1) $$P(\%)=\frac{W-D}{V}\times 100$$

where *W* (g), is the saturated mass of clay sample (the sample immersed in boiling water for 2 h), *D* (g), is the dried mass of clay sample (the sample dried in the oven at 110 °C for 48 h) and *V*(cm^3^) is the exterior (total) volume of the sample (*V* = *W* × *S*), where *S* (g), is the suspended weight of sample in water.

**Figure 2:**
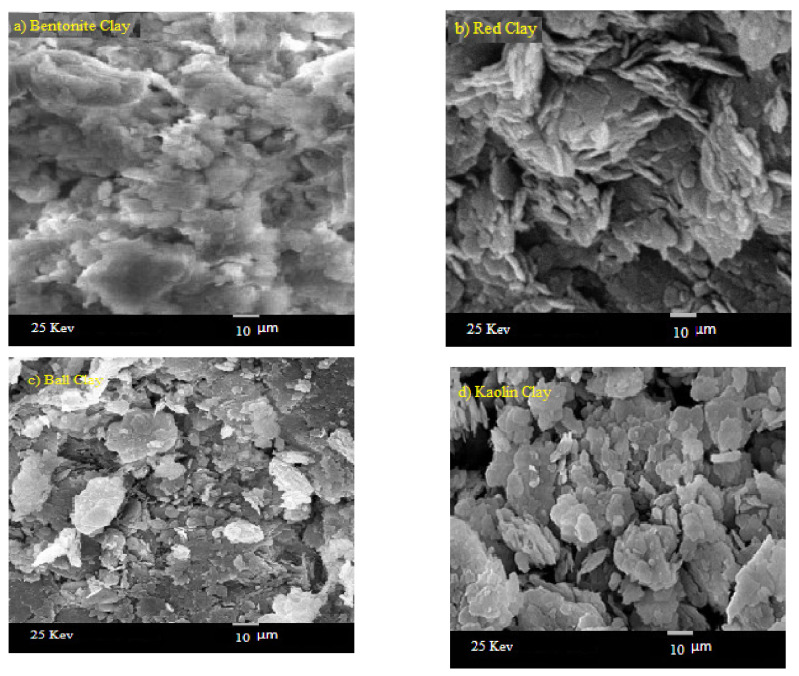
SEM images for four the different clays discussed in the present work (**a**) bentonite clay, (**b**) red clay, (**c**) ball clay (**d**) kaolin clay.

**Figure 3:**
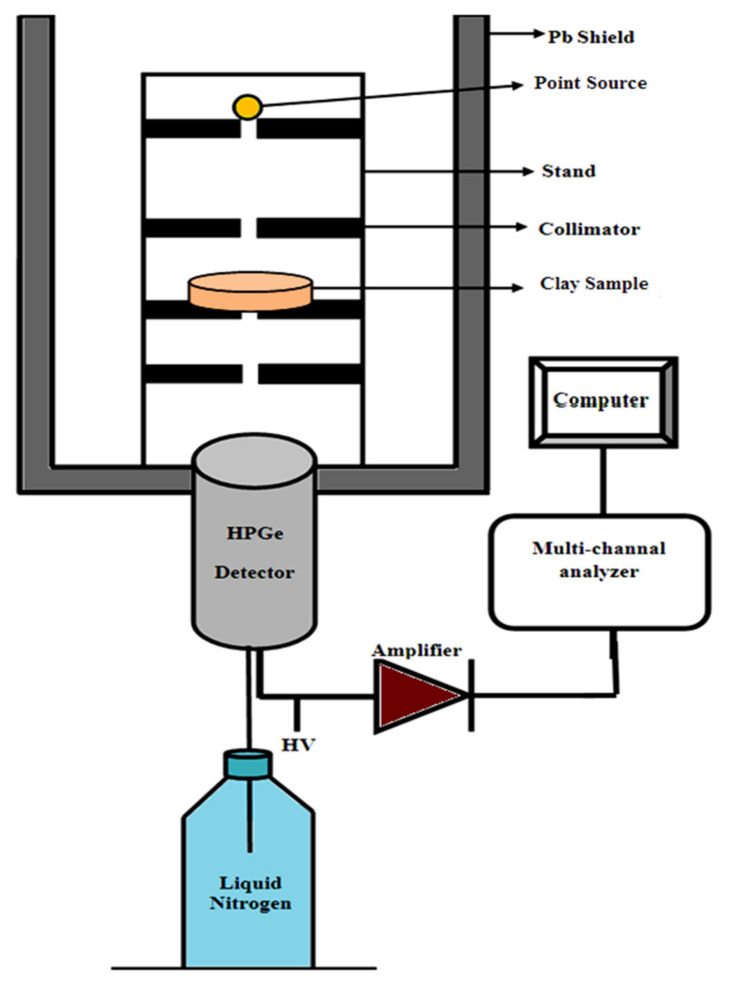
The schematic diagram of the setup used in the present work.

A scanning electron microscope (SEM, was used to show the distribution of particle inside each clay type as shown in Figure 2. To determine the elemental compositions of these clays, energy dispersive X-ray (EDX) analysis was used. The compositions are tabulated in Table 1. By knowing these compositions, the MAC can be calculated theoretically using the WinXCom program [25,26,27]. On the other hand, to calculate the MAC experimentally, the HPGe detector and three point sources of different energies were used. The sample was placed between the source and the detector as shown in Figure 3 and the measurement was done for a sufficient time so that the statistical uncertainty of the area under the peak was less than 1% and the counting rate was calculated in the presence and absence of the sample. The *MAC* is calculated according to the following equation [28,29,30]:

(2) $$MAC=\frac{-1}{x.\rho }\ln\frac{A}{A_{o}}$$

where, *A* and *A* o represent the areas under the peak or the count rates obtained from the spectrum in presence and absence of the absorbing sample respectively, *x* (cm), the thickness of the measured clay sample and *ρ* (g/cm^3^) the density. The linear attenuation coefficient or LAC defined as the probability of photons with matter per unit path length and was calculated to determine other important shielding parameters (such as HVL and TVL) where the LAC equal MAC*ρ. The HVL and TVL represent the thickness needed to attenuate 50% and 90% of initial photon intensity, respectively, and can be evaluated by the following equations [31,32]:

(3) $$\mathrm{HVL}=\frac{\ln2}{\mathrm{LAC}}\;,\;\mathrm{TVL}=\frac{\ln10}{\mathrm{LAC}}$$

The radiation protection efficiency (RPE) was determined for the studied clays to show the most efficient clay from the following equation [33].

(4) $$\mathrm{RPE}=\frac{I_{o}-I}{I_{o}}\times 100$$

## 3. Results and Discussion

Table 2 lists the experimental and theoretical mass attenuation coefficients (MAC) at four selected energies (energies emitted from Am-241, Cs-137, and Co-60) for the four investigated clays. The percent difference between the theoretical and experimental values was also determined. For example, the theoretical *MAC* value of ball clay at 0.060 MeV calculated from the XCOM software is equal to 0.287 cm^2^/g while the experimental value is equal to 0.282 ± 0.0062 cm^2^/g. These values have a relative difference of 1.54%. Meanwhile, kaolin clay has a theoretical MAC of 0.276 at 0.060 MeV and an experimental MAC of 0.270 ± 0.0055, or a R.D% of 2.05%. The highest overall MAC R.D (%) is equal to −3.02%, but most of the other results are much smaller than this value, proving that the experimental and theoretical data greatly agree with each other. This step is important to determine that the experimental data is correct and that the experimental setup used is suitable to determine the MAC of the tested clay samples.

**Figure 4:**
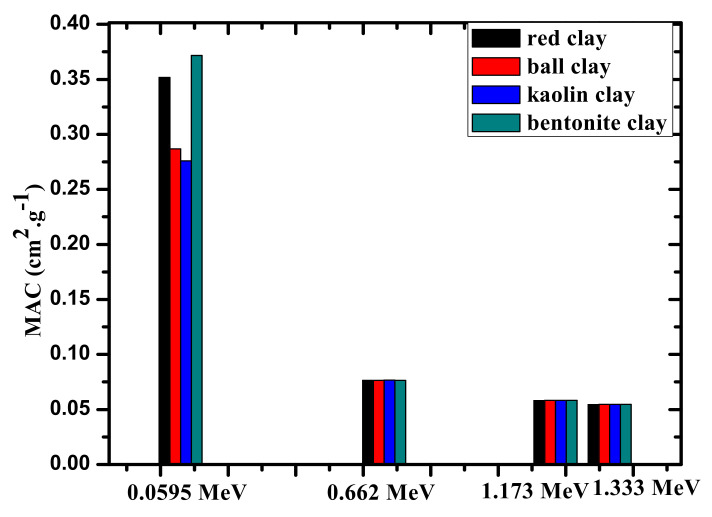
The experimental MAC values for the four investigated clay samples at four selected energies.

Figure 4 demonstrates the experimental MAC values for the four investigated clay samples at four selected energies. At the lowest tested energy, the difference between the MAC values is very clearly noticeable, while at higher energies the MACs are almost equal to each other. More specifically, at 0.060 MeV, the MAC values are equal to 0.346, 0.282, 0.270, and 0.383 cm^2^/g for red-clay, ball clay, kaolin clay, and bentonite-clay, respectively. This result shows that the shielding abilities of the clay samples at 0.060 MeV follow the order of: bentonite > red > ball > kaolin. At higher energies, this trend remains; however, the difference between the values is much smaller, meaning that the shielding capability at higher energies is practically identical. Thus, at low energies, the bentonite clay sample provides the most effective attenuation capability of the tested clays.

**Figure 5:**
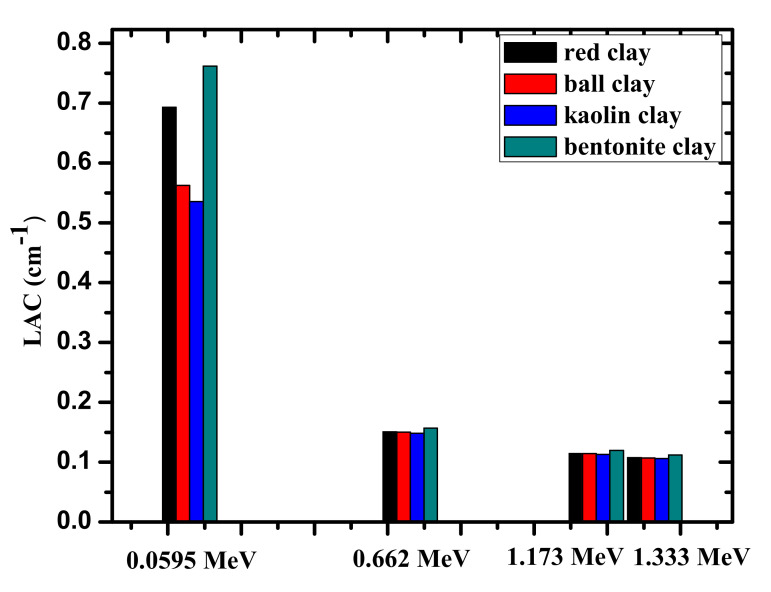
The experimental LAC values for the four investigated clay samples at four selected energies.

The linear attenuation coefficients (LACs) of the four investigated clay samples are plotted in Figure 5 at the four selected energies. Like the MAC, the difference between the LAC values is noticeable at low energies, but as energy increases, the values approach each other, resulting for all the samples in almost identical LACs. Bentonite clay has the greatest LAC at all four energies due to its high density due to its high FeO and CaO content. Meanwhile, kaolin and ball clays have the same density, but ball clay has a higher LAC than kaolin clay because it contains a larger amount of FeO and SiO_2_.

**Figure 6:**
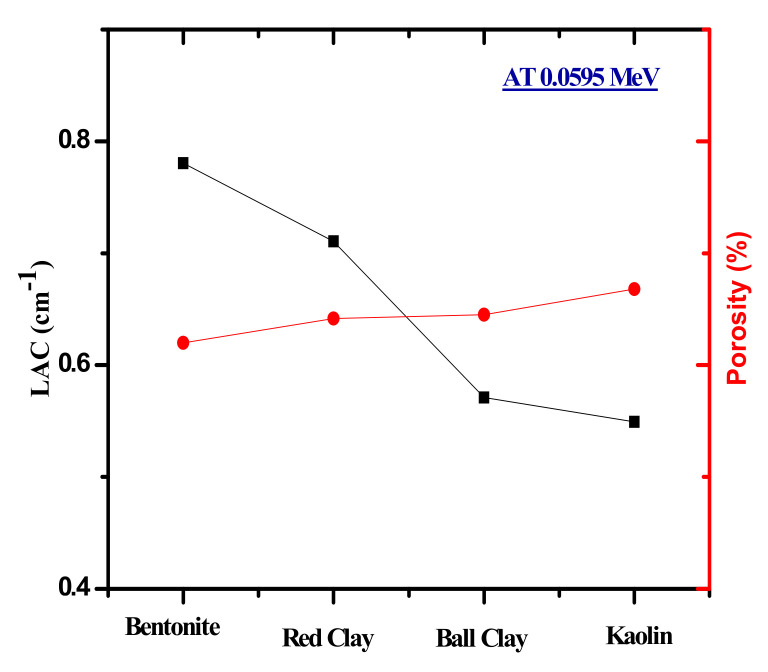
The porosity as a function of clay samples as well as the LAC values for the four investigated clay at 0.060 MeV.

**Figure 7:**
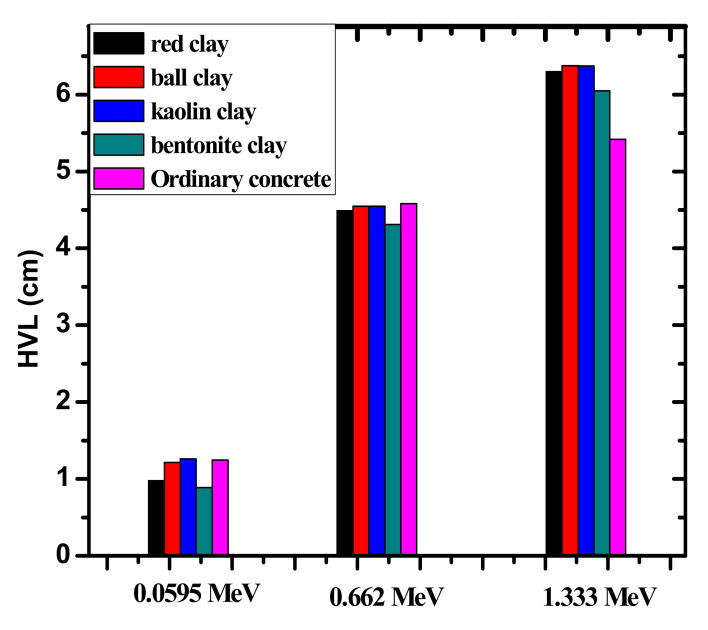
The experimental HVL values for the four investigated clay compared with ordinary concrete samples at three selected energies.

The porosity of the four clays was calculated and the results indicate that bentonite clay has the lowest level of porosity, while kaolin clay has the highest. This is reflected in the LAC, as it turns out that the one with the lowest porosity has the highest attenuation coefficient as shown in Figure 6. Figure 7 presents the half value layer (HVL) of the clay shields at low, medium, and high energies, alongside the HVL of ordinary concrete for comparison. The figure shows that the HVL increases as the energy increases. For example, the HVL of red clay increases from 0.9754 to 4.4896 cm and to 6.2973 cm at 0.060, 0.662, and 1.333 MeV, respectively, while kaolin clay has HVL values of 1.2620, 4.5466, and 6.3724 cm for the same respective energies. This result suggests than only a thin clay sample is needed to sufficiently absorb the radiation at low energies, while at higher energies a thicker sample is needed to shield the same amount of high energy radiation. More specifically, at 0.060 MeV, bentonite clay has an HVL equal to 0.8880 cm and the kaolin clay has an HVL of 1.2620 cm. This result shows that the bentonite clay is the most suitable for radiation shielding applications at low energies (below 100 keV). 

When comparing the ordinary concrete reported in [34] with the clay samples as shown in Table 3, it can be seen that at 0.060 MeV, the HVL for ordinary concrete is almost the same as the HVL for Kaolin clay (1.2469 vs 1.2620 cm) and is higher than bentonite clay (0.8880 cm) and red clay (0.9754 cm). As energy increases to 0.662 MeV, however, the HVL for ordinary concrete is almost the same as kaolin and red clay (4.5813, 4.5466, and 4.4896 cm, respectively). Lastly, at 1.333 MeV, the ordinary concrete outperforms all four of the tested clays. Thus, the figure proves that the investigated samples have a comparable shielding ability to other common radiation shields, demonstrating their potential in shielding applications. In Table 3**,** the present data were compared with the previous works reported in [21] and [22]. The results showed superiority in the current work for the attenuation coefficients at low and high energies.

**Figure 8:**
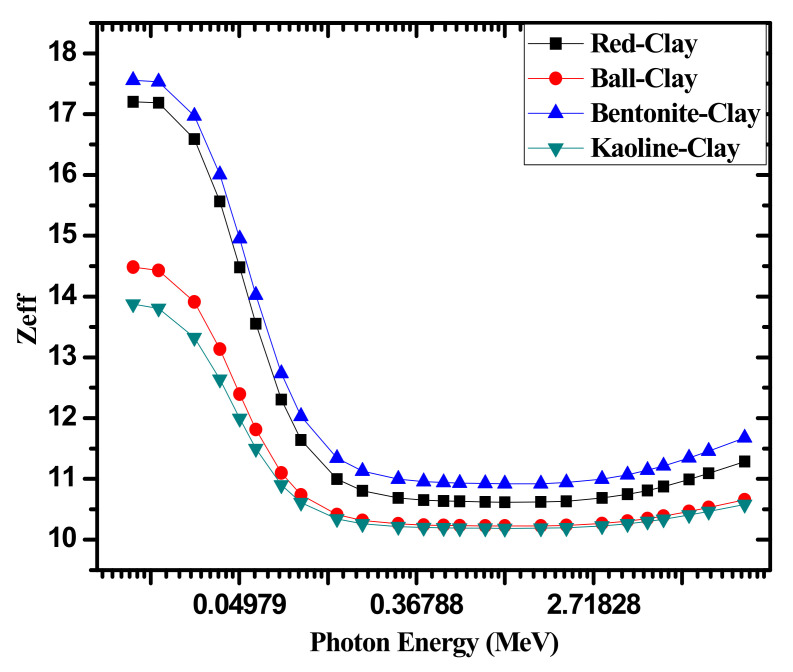
The effective atomic number Z_eff_ values for the four investigated clay samples at different energies.

The effective atomic number, or Z_eff_, (equation given in [35]) of the four investigated clay samples are graphed against increasing photon energy in Figure 8. At very low energies, Z_eff_ can be observed to sharply drop as energy increases, which can be attributed to the large inverse correlation between the photoelectric effect and energy. In the middle energy range, Z_eff_’s descent greatly slows down as the Compton scattering process takes over, and lastly as energy increases past 1 MeV, the pair production process causes Z_eff_ to increase with increasing photon energy. The figure also reveals that at all tested energies, bentonite clay has the greatest Z_eff_, followed by red clay, then ball clay, and lastly kaolin clay. Bentonite clay has the highest Z_eff_ which can be attributed to its greater concentration of Ca and S oxides, as opposed to Si, which have slightly greater atomic numbers than the metal oxides of the other samples. By that same logic, the kaolin clay has high amounts of Mg, Al, and Si compared to the other clays, which have relatively low atomic numbers, which lower its overall Z_eff_. Therefore, based on the figure, it can be concluded that bentonite clay has the greatest potential for radiation shielding applications.

**Figure 9:**
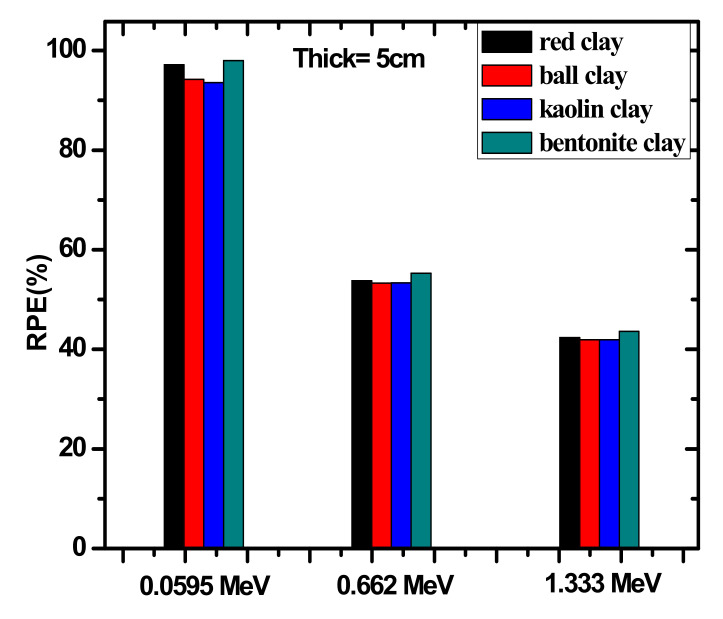
The experimental RPE values for the four investigated clay samples at three selected energies.

Figure 9 represents the radiation protection efficient, or RPE (%), of 5 cm thick samples of the tested clays against increasing energy. The figure reveals that in the three illustrated energies the RPE values follow the order of: bentonite clay > red clay > ball clay > kaolin clay. For example, at 0.060 MeV, the RPE values are equal to 97.982%, 97.137%, 94.242%, and 93.583% for bentonite clay, red clay, ball clay, and kaolin clay, respectively. This trend reveals that at this energy, the four clay samples can absorb almost all of the incoming photons, but the bentonite clay has the greatest attenuation capability of this energy, while kaolin clay has the least. In addition, as energy increases, RPE greatly begins to decrease. For instance, ball clay’s RPE decreases to 53.325% at 0.662 MeV and to 41.933% at 1.330 MeV. The decrease in value indicates that clays are much more effective at absorbing incoming photons at lower energies, while at higher energies the thickness of the samples must be increased to provide the same levels of attenuation.

## 4. Conclusions

We aimed in this experimental work to report the radiation attenuation factors for four different clays (red, ball, kaolin and bentonite clays) at certain defined energies (emitted from Am-241, Cs-137, and Co-60). The relative difference of the experimental and XCOM results in the MAC are small and confirmed the experimental setup. At 0.060 MeV, the MAC data demonstrated that the shielding abilities of the chosen clay samples follow the order of: bentonite > red > ball > kaolin. This proved that at low energies, bentonite clay sample provides the most effective attenuation capability out of the tested clays. The HVL results indicated that only a thin clay sample is needed to sufficiently absorb the radiation at low energies, while at higher energies a thicker sample is needed to shield the same quantity of high energy radiated. The Z_eff_ results also reveal that, e bentonite clay has the greatest Z_eff_, followed by red clay, then ball clay, and lastly kaolin clay. Therefore, based on the Z_eff_ parameter, it can be concluded that the bentonite clay has the greatest potential for radiation shielding applications. The RPE values at 0.060 MeV are equal to 97.982%, 97.137%, 94.242%, and 93.583% for bentonite clay, red clay, ball clay, and kaolin clay, respectively.

## Figures and Tables

**Table 1:** The chemical compositions of four different clays and their density.

Sample	Red Clay 2.02 g/cm^3^	Ball Clay 1.99 g/cm^3^	Bentonite 2.1 g/cm^3^	Kaolin 1.99 g/cm^3^
Na_2_O	0	0	1.3 ± 0.11	0
MgO	0.89 ± 0.32	0	1.18 ± 0.21	2.99 ± 0.16
Al_2_O_3_	27.34 ± 0.24	35.08 ± 0.21	20.35 ± 0.14	35.53 ± 0.24
SiO_2_	55.95 ± 0.11	58.26 ± 0.21	49.65 ± 0.22	55.26 ± 0.11
SO_3_	0	0	1.96 ± 0.46	0
K_2_O	0.98 ± 0.25	0	1.28 ± 0.13	0
CaO	0	0	10.92 ± 0.25	1.24 ± 0.42
TiO_2_	2.43 ± 0.41	2.5 ± 0.21	2.74 ± 0.14	2.76 ± 0.25
FeO	12.41 ± 0.02	4.16 ± 0.21	10.62 ± 0.19	2.22 ± 0.17

**Table 2:** The experimental and theoretical MAC by XCOM program as well as the relative deviation between the two results.

Clay Type	Nuclide	Energy (MeV)	MAC (cm^2^.g^−1^)		R.D (∆%)
			XCOM	Experimental	
red clay	Am (241)	0.060	0.352	0.346 ± 0.006	1.55
	Cs (137)	0.662	0.076	0.077 ± 0.007	−2.01
	Co (60)	1.172	0.058	0.056 ± 0.001	1.95
		1.333	0.054	0.053 ± 0.005	0.98
ball clay	Am (241)	0.060	0.287	0.282 ± 0.006	1.54
	Cs (137)	0.662	0.077	0.076 ± 0.001	1.22
	Co (60)	1.172	0.058	0.058 ± 0.005	−0.88
		1.333	0.055	0.054 ± 0.004	1.11
kaolin clay	Am (241)	0.060	0.276	0.270 ± 0.005	2.05
	Cs (137)	0.662	0.077	0.075 ± 0.005	1.95
	Co (60)	1.172	0.058	0.059 ± 0.007	−2.85
		1.333	0.055	0.054 ± 0.001	1.66
bentonite clay	Am (241)	0.060	0.372	0.383 ± 0.006	−3.02
	Cs (137)	0.662	0.077	0.078 ± 0.002	−1.55
	Co (60)	1.172	0.058	0.057 ± 0.005	0.88
		1.333	0.055	0.054 ± 0.006	0.78

**Table 3:** The shielding parameters of four clays compared with previously published data.

Attenuation Parameters	Energy (MeV)	This Work				[21]	[22]		[34]
		Bentonite Clay	Red Clay	Ball Clay	Kaolin Clay	Bentonite/Cement	Ball Clay	Kaolin Clay	Ordinary Concrete
LAC	0.060	0.7806	0.7106	0.5709	0.5492	0.5911	0.5631	0.5421	0.5559
	0.662	0.1608	0.1544	0.1524	0.1525	0.1511	0.1513	0.1504	0.1513
	1.170	0.1224	0.1175	0.1161	0.1161	0.1241	0.1196	0.1196	0.1366
	1.330	0.1146	0.1101	0.1087	0.1088	0.1161	0.1124	0.1124	0.1279
HVL	0.060	0.8880	0.9754	1.2141	1.2620	1.1726	1.231	1.279	1.2469
	0.662	4.3101	4.4896	4.5484	4.5466	4.5873	4.581	4.609	4.5813
	1.170	5.6645	5.8990	5.9725	5.9694	5.5854	5.796	5.796	5.0743
	1.330	6.0475	6.2973	6.3759	6.3724	5.9703	6.167	6.167	5.4194
MFP	0.060	1.2811	1.4072	1.7515	1.8207	1.6918	1.776	1.845	1.7989
	0.662	6.2182	6.4772	6.5619	6.5594	6.6181	6.609	6.649	6.6094
	1.170	8.1721	8.5104	8.6165	8.6120	8.0580	8.361	8.361	7.3206
	1.330	8.7246	9.0851	9.1985	9.1934	8.6133	8.897	8.897	7.8186
TVL	0.060	2.9499	3.2402	4.0330	4.1923	3.8954	4.089	4.248	4.1421
	0.662	14.3180	14.9142	15.1094	15.1035	15.2388	15.219	15.310	15.2187
	1.170	18.8170	19.5959	19.8402	19.8300	18.5543	19.252	19.252	16.8564
	1.330	20.0892	20.9193	21.1803	21.1686	19.8328	20.486	20.486	18.0030

## Data Availability

All data are available in the manuscript.mdpi.com/ethics. You might choose to exclude this statement if the study did not report any data.
